# Agentic markets

**DOI:** 10.1007/s12525-026-00906-y

**Published:** 2026-06-06

**Authors:** Martin Bichler

**Affiliations:** Department of Computer Science, Technical University of Munich, Boltzmannstr. 3, Garching, Germany

**Keywords:** AI agents, Markets, Search frictions, Signal dilution, D83, L13, D47

## Abstract

Generative AI enables autonomous software agents that can search, compare, and transact across digital marketplaces, promising large reductions in consumer search costs and improved matching between buyers and sellers. This paper argues that such gains are not automatic. Drawing on economic search theory, we first discuss the impact of reduced search costs on markets. Then, we show how the behavior of current AI agents introduces frictions that limit competitive outcomes. Empirical studies reveal persistent deviations of AI agents from optimal search behavior that function as behavioral search costs even when technical search costs approach zero. At the same time, AI-generated content contributes to signal dilution, reducing the informativeness of offers by AI agents and weakening effective product differentiation. These forces interact with low entry costs, which encourage excessive and often low-value entry. Together, they can trap agentic markets in inefficient equilibria. We outline key implications for electronic marketplaces and highlight promising directions for future research on agentic markets.

## Introduction

Generative artificial intelligence (GenAI) has enabled the emergence of autonomous software agents that can search, compare, negotiate, and transact in digital environments. These *AI agents*, typically built on large language models (LLMs), interpret natural-language objectives and execute multi-step economic tasks with limited human intervention. As such agents increasingly act on behalf of buyers and sellers in electronic marketplaces, they fundamentally alter how market interactions are organized.

We refer to such settings as *agentic markets*: electronic markets in which autonomous AI agents act on behalf of buyers and/or sellers to execute search, evaluation, negotiation, and transaction decisions. In this paper, a *market* is understood as a decentralized electronic exchange environment in which prices, search, and matching determine trade outcomes. A *trade* denotes the matching and transaction outcome resulting from these search and pricing processes.

The central question we address is: *How does delegation to AI agents alter equilibrium prices, entry, and matching efficiency in markets characterized by search frictions and product differentiation?*

We argue that agentic markets do not eliminate market frictions; they transform them. While AI agents can drastically reduce *technical* search costs, delegation to probabilistic, content-generating systems introduces new *behavioral* and *informational* frictions. Rather than converging toward frictionless competitive equilibria, agentic markets may sustain price dispersion, inefficient entry, and matching distortions through transformed economic primitives. These arguments challenge the common presumption that AI-mediated markets necessarily approximate Bertrand-style competition as search costs approach zero.

### Research gap and positioning

Existing research does not yet provide a unified economic framework for analyzing agentic markets. Classical multi-agent systems research studies coordination and mechanism design among engineered, usually rule-based agents operating under well-defined strategic rules (Van der Hoek & Wooldridge, 2008; Shoham, 2008). This literature does not consider probabilistic, content-generating LLM-based agents whose behavior may depend strongly on model architecture, prompting, and interface design.

The literature on algorithmic pricing and collusion analyzes learning dynamics among seller-side pricing algorithms (Bichler et al., 2025; Fish et al., 2024). While highly relevant, this work focuses primarily on strategic price adjustment and does not analyze markets in which search, evaluation, and matching decisions are delegated to generative agents on both sides of the market.

At the same time, emerging empirical studies document recurring but model-dependent behavioral patterns of LLM-based agents—including order effects, limited exploration, hallucinations, and signal compression (Bansal et al., 2025; Wang et al., 2025b; Nejad et al., 2025; Ren et al., 2025). However, these findings remain largely disconnected from established microeconomic models of search and product differentiation.

The research gap addressed in this paper lies at the intersection of these streams: there is currently no coherent microeconomic framework that maps empirically observed LLM-agent behaviors into classical models of search, differentiation, and entry to derive equilibrium implications for prices and welfare.

### Scope

Our focus is on equilibrium implications of AI-mediated search, matching, and entry in decentralized electronic markets. We reinterpret empirically documented agent behaviors within an established search-and-differentiation framework to clarify how agentic capabilities reshape core economic primitives.

### Analytical approach and contribution

To analyze agentic markets, we build on the Bertrand–Chamberlin–Diamond tradition as formalized by Anderson and Renault (1999), which integrates search costs, product differentiation, and endogenous entry in oligopolistic markets. This framework is particularly suited to our objective because it explicitly models the economic primitives that AI agents affect: search costs, information precision, and entry incentives. We show how:

- Model-dependent order effects, limited exploration, and related agent behaviors can act as residual search frictions that may persist even under technological improvement.
- Signal dilution from AI-generated content can be interpreted as noise in match-value signals, reducing effective differentiation.
- Lower entry costs induced by AI-enabled content generation can exacerbate excessive entry and low-value product proliferation.

In addition, vulnerabilities such as hallucinations, prompt injection, and information leakage translate into market-level frictions. Rather than representing isolated technical failures, these phenomena introduce reliability risk, stochastic pricing deviations, and distortions in strategic transparency that affect equilibrium outcomes.

By systematically mapping technological features of AI agents to shifts in economic parameters, we provide a conceptual bridge between emerging empirical findings on AI agents and established microeconomic theory. Table 2 summarizes this mapping.

Our contribution goes beyond reinterpreting parameters in an existing search model. In agentic markets, the key primitives of search and differentiation become *endogenous* to system design choices: ranking algorithms, interface constraints, verification regimes, and data portability rules determine (i) which alternatives are sampled, (ii) how match signals are constructed and compressed, and (iii) how entry translates into visibility and demand. This endogeneity implies that equilibrium outcomes are shaped as much by platform architecture as by consumer preferences and technology. We therefore treat market design and governance levers as part of the economic environment, not as an afterthought.

Beyond price competition, agentic intermediation reshapes platform-level control over visibility and transaction routing. When AI agents mediate discovery and execution within platform-controlled environments, intermediation power shifts from decentralized consumer search to algorithmic gatekeeping. This reinforces the need for a rigorous economic framework to understand the consequences of AI-mediated exchange.

Overall, this Foundations article makes three contributions. First, it provides a precise conceptual definition of agentic markets and delineates their scope. Second, it integrates recent empirical findings on AI-agent behavior into a well-established framework of search and differentiation. Third, it derives implications for platform design and governance aimed at mitigating inefficient equilibria in markets increasingly mediated by AI agents.

### Methodological approach

As a Foundations article, this paper develops a conceptual synthesis of emerging research on AI-agent behavior and its economic implications. Our objective is not to conduct a systematic review in the narrow methodological sense, but to identify recurring empirical regularities and integrate them into a coherent microeconomic framework.

To this end, we conducted a structured literature search covering the period 2023–2025, reflecting the rapid emergence of LLM-based agents in market contexts. We searched major academic databases (Google Scholar, SSRN, arXiv, and AIS eLibrary) and screened proceedings of leading conferences in artificial intelligence and information systems (e.g., AAAI, ICML, ICIS, NeurIPS), as well as relevant economics and IS journals. Search terms included combinations of “LLM agents,” “agentic markets,” “AI bias,” “algorithmic pricing,” “AI negotiation,” “signal distortion,” “hallucinations,” and “market design.”

We included empirical, experimental, and simulation-based studies that analyze the behavior of LLM-based agents in economically relevant decision environments, such as search, ranking, pricing, negotiation, or matching tasks. Conceptual papers without behavioral evidence and purely technical performance benchmarks unrelated to market interaction were excluded.

Selected studies were then analyzed through a mechanism-based coding approach. Specifically, we identified recurring behavioral patterns (e.g., position bias, early stopping, signal compression, stochastic negotiation behavior) and mapped these patterns to economically meaningful primitives within established models of search, differentiation, and entry. The goal of this synthesis is not exhaustiveness, but theoretical integration: to translate documented agent behaviors into shifts in core economic parameters such as search costs, signal precision, and entry costs. This approach should ensure that the conceptual framework developed in this paper is grounded in documented empirical regularities rather than isolated case examples.

## Capabilities of generative AI–based agents

We identify five capabilities of GenAI-based agents that are economically significant for electronic markets. These capabilities modify core primitives of standard microeconomic models and therefore shape equilibrium outcomes in agentic markets.

Unlike earlier rule-based or retrieval systems, GenAI-based agents combine natural-language interpretation, probabilistic reasoning, and tool execution within a unified decision-making architecture. Rather than optimizing over fully specified utility functions, these agents infer latent preferences from loosely articulated goals, process large sets of alternatives, transform informational signals, and autonomously execute transactions.

Industry deployments illustrate this shift. For example, conversational shopping systems such as Google’s Gemini Enterprise or ChatGPT-based purchasing interfaces allow users to delegate search and execution to an AI agent that interprets objectives, prioritizes alternatives, and completes transactions within a single mediated interaction. Similar systems are emerging in dynamic pricing and procurement contexts (Wang et al., 2025a). While the specific implementations vary, the economically relevant capabilities are structurally similar.

Note that buying transactions can involve bundles of goods or services to be purchased. The travel industry provides illustrative use cases, where humans specify high-level objectives in natural language (e.g., “Book a trip and hotel in Munich for next Tuesday”), and autonomous agents decompose these requests, identify counterpart agents, negotiate terms, and execute bookings end-to-end.^1^ Let us now discuss which features of AI agents are particularly useful on markets.

1. First, autonomous delegated search allows agents to *interpret loosely specified natural-language objectives*. Rather than operating on fully specified utility functions, AI agents infer latent preferences, which might also introduce potential noise in match evaluation. This replaces explicit preference revelation with probabilistic inference.
2. Second, they can *inspect large numbers of alternative offerings*. This reduces technical search costs and shifts the locus of search from human cognition to algorithmic mediation.
3. Third, AI agents can *summarize and compare product information*. While this can reduce information overload, it may also compress or distort match-value signals, affecting effective product differentiation.
4. Fourth, *Automated negotiation and execution* allow agents to initiate transactions, trigger renegotiations, and respond dynamically to price changes. This creates the possibility of rapid strategic interaction and continuous price adaptation.
5. Fifth, *low-cost content and offer generation* reduces barriers to entry by lowering the cost of creating listings, product variants, or digital goods. This can increase variety but may also induce excessive entry and low-value proliferation.

**Table 1:** Capabilities of generative AI-based agents and their market-level implications

Capability	Economic effect	Potential market-level threat
*Preference interpretation under natural language*	Infers latent user preferences from loosely specified goals; partially constructs utility representations	Preference inference noise; mismatch between inferred and true utility; distorted matching outcomes
*Autonomous delegated search across platforms*	Reduces technical search costs; shifts search from human cognition to algorithmic mediation	Residual exploration frictions arising from model-dependent order effects, limited exploration, or early stopping; persistent price dispersion
*Summarization and comparison of product information*	Aggregates and transforms product signals; reduces information overload	Signal compression and distortion; reduced effective differentiation; noisier match-value signals
*Automated negotiation and execution*	Enables rapid transaction initiation and dynamic renegotiation; allows near-continuous price adaptation	Reliability and strategic noise; stochastic pricing deviations; unstable bargaining outcomes or volatility
*Low-cost content and offer generation*	Reduces entry barriers; increases variety and listing volume	Excessive entry; low-value proliferation; welfare losses from weakly differentiated offerings

Each capability in Table 1 modifies a distinct primitive in standard microeconomic models. Preference interpretation affects the representation of utility; delegated search alters effective search costs *c*; summarization and signal transformation affect informational precision and effective differentiation; automated negotiation changes the dynamics of strategic adjustment; and low-cost content generation lowers entry costs.

While these capabilities can reduce technical transaction costs, they simultaneously introduce new sources of behavioral bias, informational noise, and strategic instability. The economic question is therefore not whether AI agents reduce frictions, but which frictions are transformed, which persist, and how these shifts alter equilibrium prices, entry, and welfare. Embedding these capabilities into established models of search, pricing, and differentiation provides the analytical foundation for answering this question.

## Economic model

Microeconomic theory provides several benchmark models that help us reason about how delegation to AI agents may reshape market outcomes. Oligopoly theory is a cornerstone of this analysis. Bertrand (1883) studied price competition with homogeneous goods and perfectly informed consumers. In the Bertrand model, even with only two firms, equilibrium prices are driven to marginal cost. This stark prediction—often referred to as the “Bertrand paradox”—suggests that if AI agents eliminate informational frictions entirely, price dispersion for homogeneous goods should disappear.

The Bertrand model, however, abstracts from search frictions and product differentiation. Agentic markets are unlikely to satisfy these assumptions. Products are differentiated, and even when technical search costs fall, residual frictions may arise from algorithmic biases and signal distortion. A framework that explicitly incorporates both search behavior and differentiation is therefore required.

Search frictions were introduced by Diamond (1971) in a model with homogeneous goods. Diamond shows that even arbitrarily small search costs generate a discontinuity: firms optimally set monopoly prices, and consumers do not search in equilibrium. The reason is that once a consumer has observed a price, the expected gain from searching another firm is smaller than the search cost. Anticipating this, firms raise prices, leading to a monopoly outcome independent of the number of firms.

Product differentiation fundamentally alters this logic. Chamberlin (1933) argued that heterogeneity in consumer preferences grants firms market power even when many firms are active. In agent-driven markets, differentiation may become highly granular, as AI agents can process high-dimensional preferences (e.g., “a house near a park with morning light”) that are difficult for human consumers to operationalize. In such environments, equilibrium prices depend not only on search costs but also on the strength of preferences for variety.

To analyze the equilibrium consequences of AI-mediated search and trade, we require a framework that jointly captures (i) consumer search frictions, (ii) product differentiation, and (iii) endogenous entry. The model of Anderson and Renault (1999) integrates search costs and product differentiation within a unified equilibrium structure and characterizes how search frictions shape prices, market structure, and entry incentives. This framework is particularly well suited to studying agentic markets, where AI agents may alter effective search costs, distort match-value signals, and lower entry barriers, thereby shifting the underlying economic primitives that determine equilibrium outcomes.

**Fig. 1:**
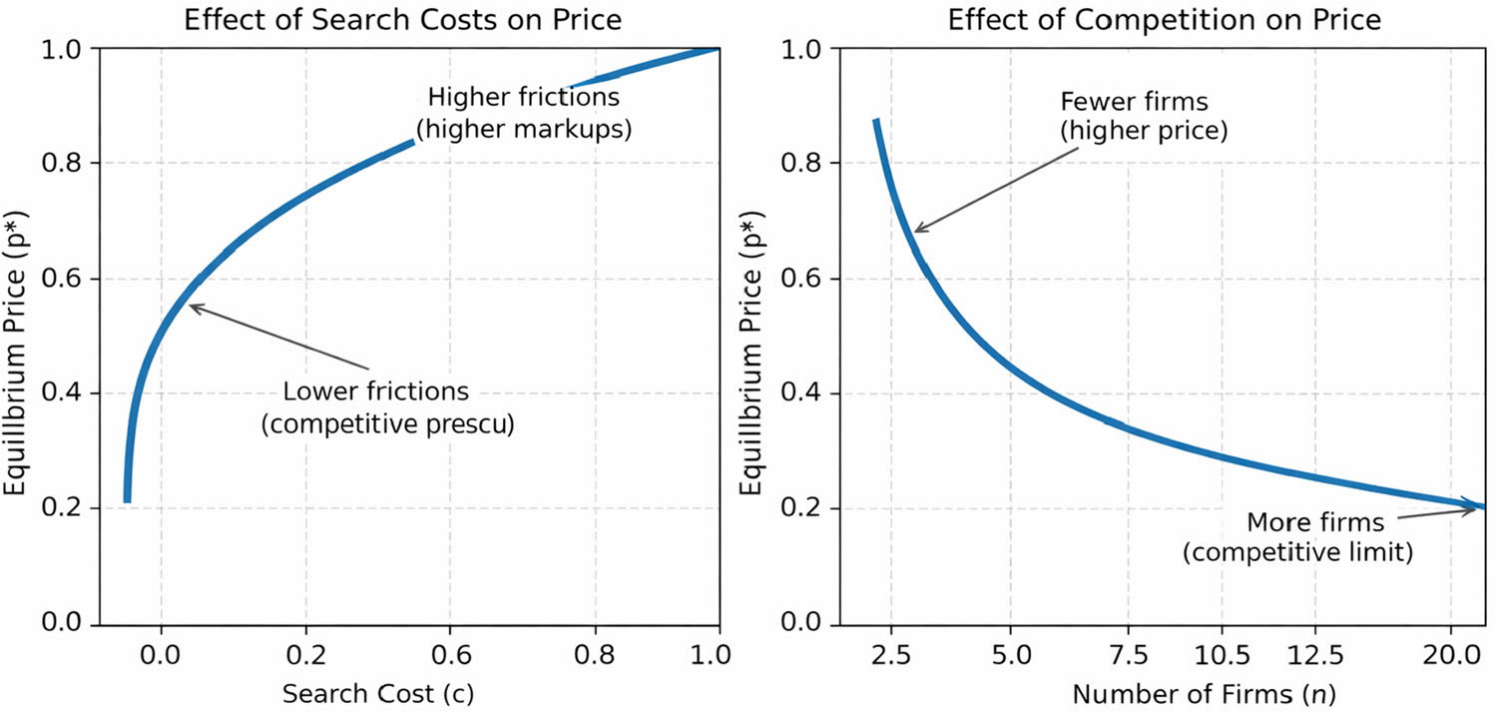
The left panel illustrates that the equilibrium price $p^*$ is strictly increasing in search costs *c*, as higher frictions reduce consumers’ reservation match values. The right panel shows that $p^*$ is strictly decreasing in the number of firms *n*, as increased competition increases the probability of consumers finding a better match elsewhere

### Markets with search costs and product differentiation

We briefly summarize the model of Anderson and Renault (1999), which serves as our baseline framework. Our aim is not to reproduce the full analysis, but to identify the core economic primitives that are later reshaped in agentic markets. The model is well suited to our purposes because it combines three ingredients that are central to our argument: costly search, product differentiation, and endogenous consumer stopping behavior. In this sense, it provides a unified bridge between the Bertrand logic of price competition, the Diamond logic of search frictions, and the Chamberlin logic of differentiated products.

**Fig. 2:**
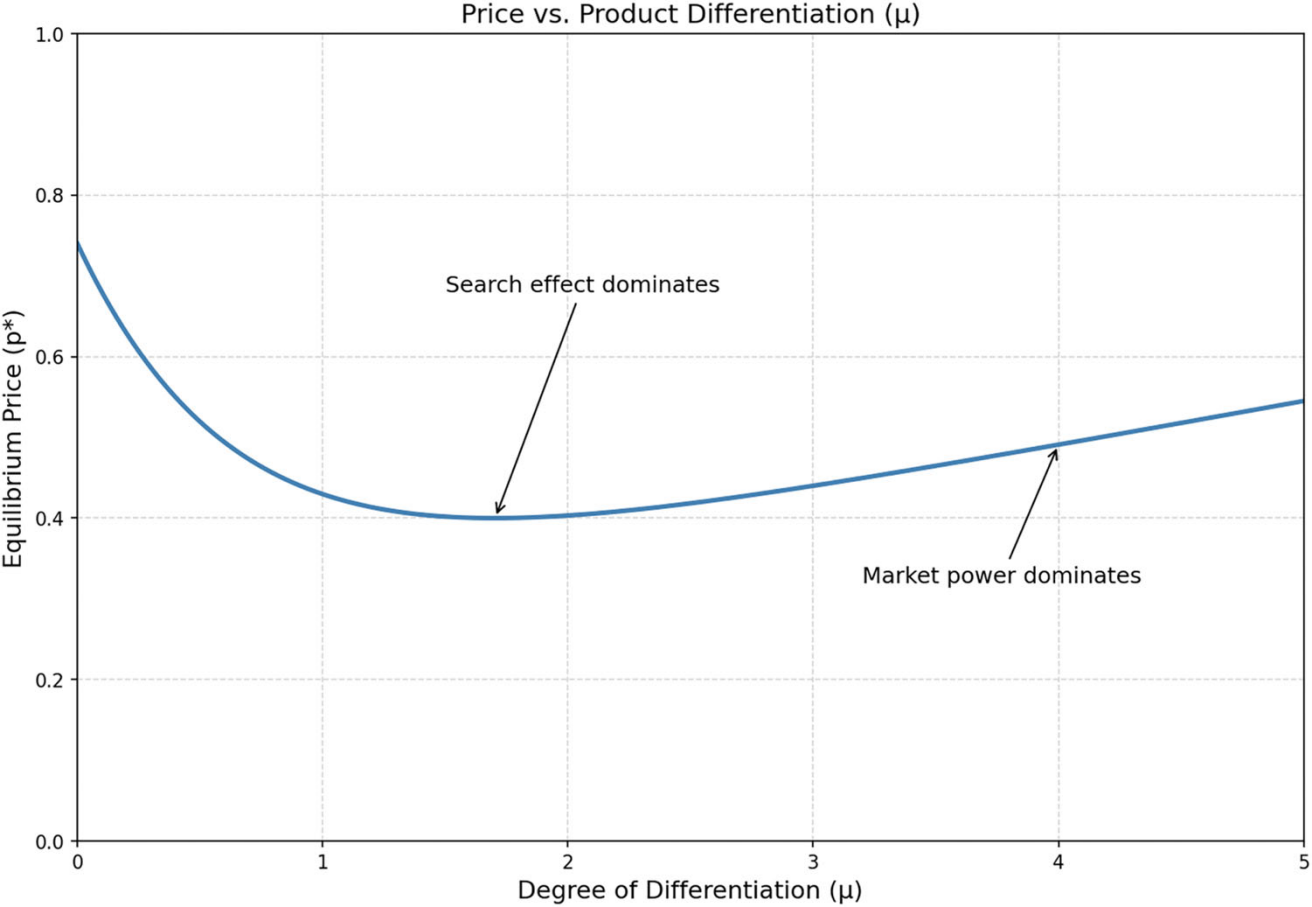
This plot illustrates that increasing differentiation initially lowers prices (by encouraging search) before raising them (due to market power)

Consider a market with *n* firms, each offering a differentiated product at zero marginal cost, and a population of *L* consumers. Consumer *l*’s utility from purchasing product *i* at price $p_i$ is

1 $$\begin{aligned} u_{li}(p_i) = -p_i + \mu \epsilon _{li}, \qquad i = 1, \dots , n, \end{aligned}$$

where $p_i$ denotes the price of product *i*. The parameter $\mu > 0$ scales the importance of product differentiation: the larger is $\mu$, the more strongly consumers value idiosyncratic product fit relative to price. The random variable $\epsilon _{li}$ captures the match value between consumer *l* and product *i*, and is drawn i.i.d. from a distribution *F* with density *f* on support [*a*, *b*).

Consumers do not observe all match values at once. Instead, they search sequentially and incur a search cost *c* each time they inspect an additional firm. If a consumer purchases product *i* after visiting *k* firms, total utility is

2 $$\begin{aligned} U = u_{li}(p_i) - kc. \end{aligned}$$

Search is therefore valuable because it may uncover a better-fitting product, but it is also costly because each additional inspection consumes resources.

Consumers follow an optimal sequential search rule. They begin by visiting one firm chosen at random. After observing the realized match value at that firm, they decide whether to stop and buy or continue searching. If they continue, they sample another firm from those not yet visited. With perfect recall, a consumer can always return to the best option encountered so far.

The key object governing this stopping decision is the expected incremental benefit from one more search. If the consumer’s current best match value is *x*, then the expected gain from searching one more firm is

3 $$\begin{aligned} \mu g(x) = \mu \int _x^\infty (\epsilon - x) f(\epsilon )\, d\epsilon . \end{aligned}$$

This expression has a natural interpretation: the consumer gains only when the next draw exceeds the best match already observed, and the gain is the improvement over that current best option. Search continues as long as this expected gain exceeds the search cost.

Accordingly, the optimal stopping rule is characterized by a reservation match value $\hat{x}$ satisfying

4 $$\begin{aligned} g(\hat{x}) = \frac{c}{\mu }. \end{aligned}$$

Consumers continue searching whenever their best observed match is below $\hat{x}$ and stop otherwise. This condition makes the comparative statics transparent: higher search costs reduce search intensity, while stronger product differentiation raises the value of finding a better match and can therefore increase search.

Given this consumer behavior, firms choose prices strategically. The model yields a symmetric equilibrium price $p^*$, derived from firms’ first-order conditions. Intuitively, equilibrium demand has two components: some consumers purchase immediately when they encounter a sufficiently attractive offer, while others continue searching and eventually return to the best firm among those visited.

Two limiting cases are especially useful for our later discussion. When product differentiation is weak relative to search costs, the model approaches the Diamond outcome: consumers do not search, and firms charge monopoly prices. By contrast, when differentiation is sufficiently strong, consumers search not only for lower prices but also for better product fit. Search then restores competitive pressure, so equilibrium prices fall below monopoly levels.

This framework, therefore, highlights the three economic primitives that are most important for our analysis of agentic markets: search cost *c*, differentiation intensity $\mu$, and entry incentives. In the sections that follow, we examine how AI-mediated search, AI-generated content, and lower entry barriers can alter precisely these primitives.

### Equilibrium prices and market structure

This active search behavior preserves competitive pressure, ensuring that equilibrium prices remain below the monopoly level as long as the reservation match value $\hat{x}$ lies strictly within the support of the match-value distribution (see Fig 1, left panel). When search costs increase, consumers search less intensively, reducing competitive pressure and raising equilibrium prices. Conversely, as the number of firms increases, the expected gain from continued search rises, intensifying competition and lowering prices (see Fig. 1, right panel).

It is interesting to look at the relationship between the equilibrium price and product diversity, $\mu$. An increase in $\mu$ generates two opposing forces. First, a direct “market power” effect increases prices because consumers have stronger preferences for their ideal product and are thus less price-sensitive. Second, an indirect “search” effect decreases prices because a higher $\mu$ increases the potential gains from finding a better match, which raises the reservation threshold $\hat{x}$ and encourages more search. For low levels of product differentiation, the search effect dominates: introducing diversity lowers prices. However, at sufficiently high levels of differentiation, the market power effect prevails, causing prices to rise (see Fig. 2).

**Fig. 3:**
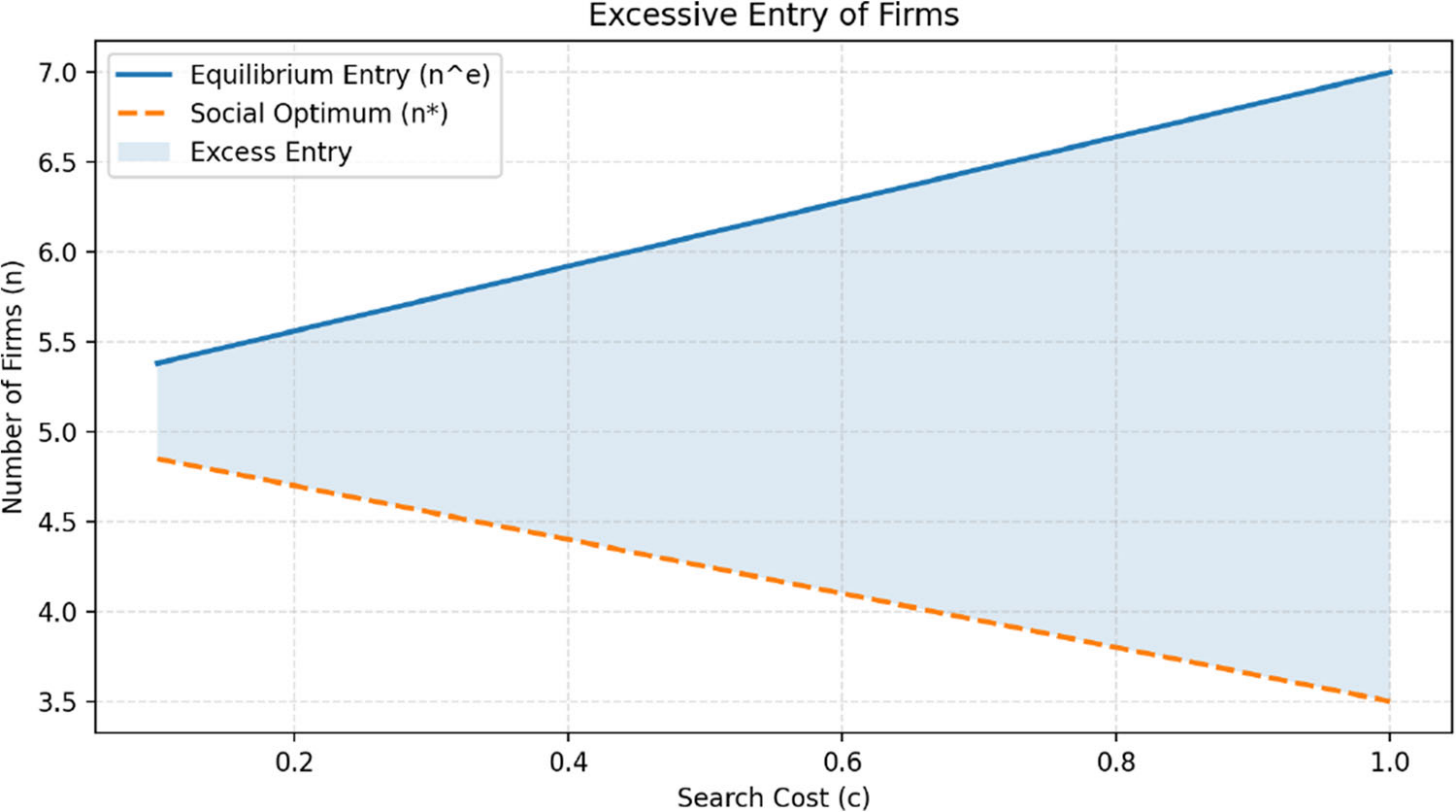
The figure visualizes the gap between the equilibrium number of firms ($n^e$) and the social optimum ($n^*$), showing how high search costs exacerbate the inefficiency of excessive entry

Another key insight by Anderson and Renault (1999) concerns market entry and whether the number of firms that enter is socially optimal. From a welfare perspective, an additional firm is desirable only if the extra match value it creates for consumers outweighs the cost of entering the market. In the model, firms decide whether to enter based solely on private profits, which include gains from attracting customers away from rivals (“business stealing”) even when the new product adds little real value. Taken together, these results show that equilibrium prices and market structure are jointly shaped by three key parameters: search cost *c*, product differentiation $\mu$, and entry conditions. Technological changes that affect any of these primitives can therefore alter both prices and welfare.

As a result, the model predicts that firms have a stronger incentive to enter than society would like. This distortion becomes more severe when search costs are high: higher search costs push up equilibrium prices and profits, encouraging even more firms to enter. Yet the presence of additional firms forces consumers to search more, reducing welfare. The equilibrium, therefore, features too many firms, and the degree of excessive entry grows with search frictions (see Fig. 3).

In summary, with vastly decreasing search costs in agentic markets, competition intensifies because consumers can easily check “one more option” to find a better deal. This intensifies competitive pressure and can reduce markups. Consumers search more exhaustively (reservation utility rises), ensuring they find the product that best fits their specific preferences rather than settling for a “good enough” option. Although there is some tendency toward excessive entry even at low search costs, in a low-friction world, the model predicts an efficient market.

The analysis so far summarizes the results of Anderson and Renault (1999). Building on this framework, we reinterpret key model parameters in light of empirical findings on AI-agent behavior. Specifically, we examine how (i) behavioral search costs induced by agent biases, (ii) signal noise reflecting AI-generated content, and (iii) reduced entry costs associated with AI-enabled product generation alter equilibrium prices, matching efficiency, and entry incentives.

## Economics of markets with AI agents

Standard models of consumer search suggest that substantial reductions in search frictions intensify competition, narrow price dispersion, and move equilibrium prices closer to competitive levels. One might therefore expect that delegating search and transaction decisions to AI agents reduces search costs sufficiently to strengthen competitive pressure and improve allocative efficiency.

However, a growing body of experimental and simulation-based evidence suggests that LLM-based agents do not always search in a fully frictionless or fully rational manner. Across a range of environments, some agents exhibit model-dependent order effects, limited exploration, early stopping, and sensitivity to prompt framing (Bansal et al., 2025). While the direction and magnitude of these behaviors vary across models and decision settings, they indicate that even when the technological cost of inspecting additional offers is negligible, residual behavioral frictions may persist and affect search depth and matching outcomes.

The objective of this section is not to test these mechanisms empirically, but to interpret documented agent behaviors within a unified economic framework and clarify their equilibrium implications in the search-and-differentiation model of Anderson and Renault (1999).

### Agentic biases as residual search frictions

Despite the potential for automation and efficiency gains, experimental evidence indicates that AI agents do not always implement fully rational search strategies. Importantly, the documented biases are not uniform across models. Allouah et al. (2025) show that ranking effects depend on the underlying LLM architecture: some agents display stronger positional preferences than others, and these preferences need not take the form of a simple bias toward the first displayed option. Similarly, Bansal et al. (2025) report that some agents exhibit order effects or degraded performance when faced with larger option sets, while the direction and magnitude of these effects vary across models and decision stages.

In the Anderson-Renault framework, consumers optimally continue searching as long as the expected gain from another draw exceeds the search cost *c*. The economically relevant implication of the evidence above is therefore not a universal “first-result bias,” but a more general tendency of some agent architectures to truncate exploration, overweight a subset of encountered offers, or terminate search prematurely relative to the frictionless benchmark. When this occurs, the probability of sampling additional alternatives declines. From the perspective of the model, the agent behaves *as if* effective search frictions remain positive, even when the technological cost of inspecting further offers is low. We capture this idea with a reduced-form decomposition:

5 $$\begin{aligned} c_{\text {effective}} = c_{\text {tech}} + c_{\text {behavior}}, \end{aligned}$$

where $c_{\text {tech}}$ captures technological search costs and $c_{\text {behavior}} \ge 0$ represents residual exploration frictions induced by agent-specific stopping rules, positional preferences, or limited consideration sets. This interpretation should be understood as a reduced-form mapping rather than a literal claim that all ranking effects are equivalent to a uniform additive search cost. The key point is that when agent behavior reduces continuation or narrows the set of sampled alternatives, equilibrium search is less extensive than in the frictionless benchmark. Even if $c_{\text {tech}} \rightarrow 0$, positive residual exploration frictions can sustain markups above the competitive benchmark and allow price dispersion to persist.

The aggregate market effect depends on the composition of agent types. If many agents in a market rely on similar architectures and exhibit systematically limited exploration, the resulting reduction in effective search can weaken competitive pressure. In more heterogeneous environments, different positional preferences may partially offset each other. However, even in such cases, market-wide exploration may remain incomplete if multiple agent types sample only a limited subset of the available alternatives.

### Signal distortion as reduced differentiation

Recent empirical work suggests that AI agents may replace search frictions with information frictions. In labor markets, Nejad et al. (2025) show that LLM assistance disproportionately improves lower-quality applications, compressing signal quality so that heterogeneous candidates appear more similar. In online gig markets, Ren et al. (2025) find that AI-generated proposals weaken the informational content of bids, leading employers to rely more heavily on coarse heuristics.

In the Anderson–Renault framework, product differentiation is governed by the dispersion of match values scaled by $\mu$. When AI-generated content homogenizes observable attributes or compresses quality signals, the informational precision of match values declines. This can be represented as

6 $$\begin{aligned} \hat{\epsilon }_{li} = \epsilon _{li} + \eta , \end{aligned}$$

where $\eta$ captures noise introduced through signal compression or content homogenization.

A reduction in effective differentiation can intensify price competition when search remains sufficiently active, as consumers perceive alternatives to be closer substitutes. However, this force need not dominate when signal dilution is combined with residual exploration frictions. If agents sample only a limited subset of available options or exhibit low continuation probabilities, weaker differentiation does not necessarily translate into stronger competitive pressure. In that case, reduced signal precision primarily lowers matching quality, while incomplete exploration preserves firms’ local market power.

The interaction between signal dilution and residual exploration frictions is therefore central. Signal compression reduces allocative efficiency by making alternatives harder to distinguish, while limited exploration reduces the likelihood that better matches are ever discovered. Together, these mechanisms may sustain equilibria characterized by mismatches, persistent markups, and excessive entry of weakly differentiated or difficult-to-verify products. More generally, the market-level effect depends on how signal distortion interacts with the distribution of agent behaviors: if agent architectures differ, some distortions may offset, but reduced informational precision will still lower welfare whenever it impairs the identification of higher-match alternatives.

**Table 2:** Synthesis of empirical findings on LLM-based agents and economic interpretation

Observed mechanism	Representative studies	Empirical regularity	Economic interpretation
Model-dependent exploration bias (e.g., order effects, limited exploration, early stopping)	Bansal et al. (2025), Allouah et al. (2025)	Some agents exhibit model-dependent order effects, limited exploration, and satisficing rather than exhaustive search	Can act as a residual exploration friction ($c_{\text {behavior}} \ge 0$), limiting effective search and preventing convergence to fully competitive price equilibria even when technical search costs approach zero
Signal dilution from AI-generated content	Nejad et al. (2025), Ren et al. (2025)	AI-generated proposals compress observable differences and reduce the informativeness of market signals	Introduces noise in match-value signals ($\hat{\epsilon }_{li} = \epsilon _{li} + \eta$), lowering effective differentiation and potentially reducing allocative efficiency
Probabilistic decision-making and hallucinations	Wang et al. (2025b), Fan et al. (2024)	Agents produce inconsistent, contextually incorrect, or manipulation-sensitive outputs	Introduces stochastic pricing or matching errors, increasing outcome dispersion, monitoring costs, and unreliability in bargaining or execution
AI-enabled reduction in entry costs	Bansal et al. (2025), industry evidence on AI-assisted content generation	Lower cost of generating product variants, listings, and proposals; proliferation of low-value offerings	Reduces fixed or marginal entry costs, potentially exacerbating excessive entry and low-value variety

### Entry costs and market structure

AI also lowers the cost of creating new product variations (e.g., AI-generated books, drop-shipping variants). This lowers the cost of entry in many markets. If AI lowers entry costs, this can lead to an explosion of low-quality variety (spam entry). Combined with “signal dilution” (consumers can’t tell good from bad), this exacerbates the excessive entry discussed earlier in the “Equilibrium prices and market structure” section. The market becomes crowded with low-quality or indistinguishable variants entering solely to capture a slice of the agents’ satisficing behavior, exacerbating the welfare loss from signal dilution.

### Beyond search: Negotiation as pricing noise

While our primary focus is on AI-mediated search, LLM-based agents are increasingly also deployed in bilateral bargaining and transaction execution. In such settings, the relevant question is not only whether agents can discover attractive offers, but also whether they can implement stable and reliable pricing or bargaining behavior. Unlike classical models of rational strategic interaction, however, LLM-based agents do not necessarily play equilibrium strategies, and their performance depends strongly on implementation details, prompting, and environmental structure (Fan et al., 2024).

Recent work evaluates LLM agents through the lens of regret minimization in repeated games (Park et al., 2024, 2025). In repeated interaction, vanishing average regret implies convergence of empirical play to the set of coarse correlated equilibria (Ahunbay, 2025; Foster & Vohra, 1999; Hart & Mas-Colell, 2003). Some LLMs exhibit sublinear regret in highly structured environments, suggesting that their average behavior can approximate no-regret dynamics. However, these findings are sensitive to task design, feedback structure, and training objectives. In more complex or weakly structured environments, regret does not systematically vanish, and decision variance remains substantial. Thus, equilibrium convergence in AI-mediated negotiation is conditional rather than automatic.

For our purposes, the main implication is that negotiation does not remove frictions; it transforms them into reliability and strategic noise. Within the Anderson–Renault framework, such deviations can be interpreted as stochastic distortions of the seller’s effective pricing rule. Hallucinations, prompt sensitivity, and manipulation susceptibility may generate noisy or unstable price responses, preventing systematic convergence to the equilibrium price $p^*$. In this sense, AI-mediated negotiation affects a different margin than search or signal quality: rather than changing how many offers are sampled or how informative they are, it introduces uncertainty into the execution of pricing and bargaining itself.

Large-scale experimental evidence further suggests that successful AI negotiation typically requires constrained action spaces and carefully engineered reasoning processes (Vaccaro et al., 2025). Absent such constraints, bargaining outcomes exhibit substantial variance. This reinforces the broader conclusion of the paper: delegation to AI agents does not automatically produce frictionless market outcomes. Instead, even when search costs fall and matching technologies improve, agentic systems may introduce additional equilibrium distortions through noisy pricing and unreliable negotiation behavior.

Taken together, residual exploration frictions, signal dilution, and negotiation-related pricing noise alter three central dimensions of agentic markets: effective search costs, informational precision, and the reliability of pricing and bargaining behavior. As a result, equilibrium outcomes need not converge to the competitive benchmark even when technological search costs approach zero. Table 2 synthesizes the documented mechanisms and their mapping into the search framework.

### Core theoretical implications (informal propositions)

To make the equilibrium implications of transformed frictions transparent, we state three informal propositions that summarize how agentic capabilities map into the search-and-differentiation framework.

#### Proposition 1

(Behavioral frictions prevent full Bertrand convergence) If agents exhibit residual behavioral frictions that reduce exploration, represented as $c_{\text {effective}} = c_{\text {tech}} + c_{\text {behavior}}$ with $c_{\text {behavior}}>0$, then equilibrium markups remain bounded away from the frictionless benchmark even as $c_{\text {tech}}\rightarrow 0$. In particular, reduced continuation probabilities sustain price dispersion whenever offer sampling is incomplete.

#### Proposition 2

(Signal dilution can reintroduce Diamond-type outcomes) If AI-generated content introduces noise in match signals, $\hat{\epsilon }_{li}=\epsilon _{li}+\eta$, and agents terminate search early (high $c_{\text {behavior}}$ or low continuation), then reduced effective differentiation does not necessarily intensify competition. Instead, the market may exhibit Diamond-like pricing patterns: weak exploration combined with noisy signals can sustain high prices and misallocation despite low technical search costs. Formally, if continuation probabilities collapse sufficiently such that the expected gain from further search is below $c_{\text {effective}}$, firms face locally inelastic demand, restoring monopoly-type pricing incentives.

#### Proposition 3

(Lower entry costs amplify excessive variety under weak verifiability) When AI lowers entry costs for listings and variants, entry increases. If verifiability is limited and signals are diluted (high signal noise $\eta$), incremental entry can become predominantly business-stealing rather than value-creating, increasing the wedge between privately optimal and socially optimal entry and reducing welfare through low-value proliferation.

These propositions highlight why agentic markets need not converge to efficient outcomes: equilibrium depends on residual exploration frictions, signal fidelity, and the mapping from entry to visibility.

### Boundary conditions and contextual variation

The magnitude and relevance of the mechanisms identified above are likely to vary across market environments. In standardized B2C markets with transparent pricing and low switching costs, behavioral search biases may play a relatively larger role than signal dilution. In contrast, in B2B markets characterized by complex contracts and bilateral negotiation, reliability frictions and manipulation susceptibility may dominate.

Similarly, the implications differ between markets for standardized goods and markets for experience or credence goods. When product attributes are easily verifiable, signal dilution primarily affects ranking efficiency. For experience goods, however, AI-generated content may significantly distort perceived differentiation, amplifying welfare losses.

Platform structure also matters. In centralized platforms with algorithmic ranking, AI agents interact with curated information environments, potentially reinforcing platform gatekeeping power. In decentralized markets, by contrast, search and entry dynamics may dominate equilibrium distortions.

These distinctions suggest that the mechanisms identified in this paper are not uniform in magnitude, but context-dependent. The framework developed here provides a lens for analyzing how agentic frictions manifest differently across market architectures rather than implying homogeneous effects.

A key contextual determinant is whether agentic search is executed in an open web environment or within a vertically integrated platform ecosystem. In the latter case, ranking logic, data access, and transaction routing can endogenously shape the sampling distribution over offers. This creates an additional channel—beyond behavioral bias and signal dilution—through which equilibrium prices and entry are affected: platform-controlled visibility can alter continuation and substitution even when technical search costs are low. We return to this mechanism in Section “Platform power and agentic gatekeeping”.

### Implications

Delegation to AI agents can increase welfare by reducing *technical* frictions: agents can inspect many offers, operationalize high-dimensional preferences, and compute comparisons at low marginal cost. In the search-and-differentiation framework, this corresponds to lower $c_{\text {tech}}$ and potentially higher effective matching efficiency when exploration is sufficiently broad and signals are reliable.

However, the same delegation introduces three equilibrium risks. First, behavioral regularities such as position bias and early stopping act as residual search frictions ($c_{\text {behavior}}>0$), limiting exploration even when inspection is cheap. This can sustain markups and price dispersion because competitive pressure depends on how many alternatives are actually sampled. Second, AI-generated content can dilute the informativeness of offers and proposals, increasing noise in perceived match values ($\eta$) and reducing allocative efficiency through mismatches. Third, AI reduces the cost of creating listings and variants, amplifying entry incentives; when signals are weak, additional entry can become predominantly business-stealing rather than value-creating, leading to low-value proliferation and welfare losses.

An important open question is whether these frictions are transitional artefacts of immature technology or structural features of agentic markets. While specific failure modes (e.g., hallucination rates) may decline with improved models, the underlying sources of distortion appear structural: probabilistic preference inference, limited exploration under attention and computation constraints, and platform-mediated sampling and routing. Consequently, even technologically advanced agentic markets need not converge to frictionless competitive equilibria; instead, they are likely to exhibit transformed and persistent frictions that reshape equilibrium prices, entry, and welfare.

## Requirements for platform design and governance

The economic mechanisms identified in this paper translate into concrete design considerations for operators of electronic marketplaces and platforms deploying AI-based search, pricing, and negotiation systems. If agentic markets transform rather than eliminate frictions, platform architecture becomes a primary determinant of equilibrium outcomes. Behavioral search frictions, signal dilution, and excessive entry dynamics are not merely technological artefacts; they are shaped by interface design, information structure, and participation rules. We highlight three actionable levers for platform design.

### Reducing residual search frictions

Even small search frictions can sustain higher prices and reduce matching efficiency. As we discussed earlier, empirical evidence suggests that AI agents exhibit position bias, early stopping behavior, and satisficing tendencies, effectively introducing residual search costs. Platforms can counteract these distortions by implementing randomized or fairness-aware ranking for agentic queries, requiring agents to retrieve and compare a minimum set of alternatives, or providing structured comparison APIs rather than free-form browsing interfaces. Such interventions operationalize the theoretical insight that reducing residual search frictions intensifies competitive pressure and improves allocative efficiency.

### Restoring signal fidelity

If AI-generated content compresses observable match-value signals, effective differentiation declines and matching efficiency deteriorates. Platforms can restore signal fidelity by strengthening the verifiability and structure of product attributes. Examples include verified structured data fields, platform-issued credentials, provenance requirements, and standardized quality certifications. By improving signal reliability, platforms reduce informational noise and enable agents to more accurately identify high-match-value alternatives.

### Auditing, explainability, and interoperability as economic design variables

In agentic markets, governance mechanisms such as auditing and explainability are not merely compliance features; they modify the economic primitives that determine equilibrium outcomes. First, auditing and transparency of ranking and routing logic affect the effective continuation probability of search: when agents (and users) can verify that recommendations reflect stated objectives rather than monetization or self-preferencing, residual behavioral frictions can be reduced (lower $c_{\text {behavior}}$). Second, explainability and structured comparison interfaces improve signal fidelity by making attributes verifiable and comparable, reducing the effective noise term $\eta$ in perceived match values. Third, interoperability and data portability mitigate endogenous switching costs created by accumulated personalization histories, preserving contestability and strengthening entry incentives for smaller sellers and rival intermediaries.

These considerations suggest a unifying view: *platform policy choices map into economic parameters*. Designing audit trails, standardized attribute schemas, and interoperable agent interfaces can therefore be understood as market design levers that move equilibria toward more competitive and efficient outcomes.

### Managing entry and variety proliferation

Lower entry costs induced by AI-enabled content generation may lead to excessive entry and low-value variety. This dynamic can reduce welfare despite lower technical search costs. Platforms may mitigate inefficient proliferation through refundable listing fees, quality-based filtering mechanisms, differentiated visibility rules, or performance-based participation requirements. These levers reflect the theoretical insight that unconstrained entry in environments with weak differentiation can undermine market efficiency.

**Fig. 4:**
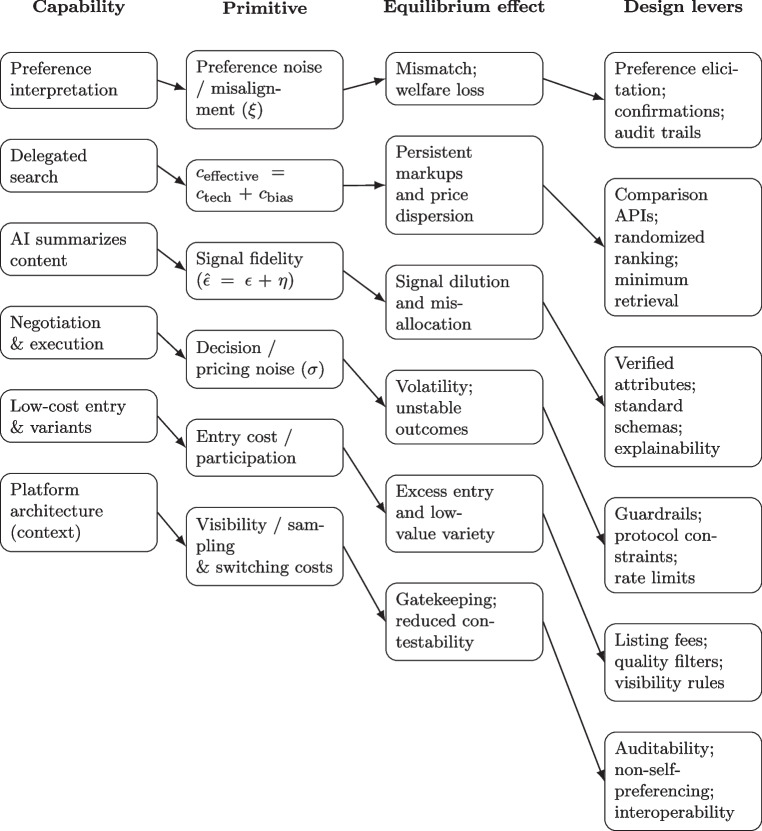
Mapping from agent capabilities (Table 1) to economic primitives, equilibrium effects, and platform design levers. The final row highlights platform architecture as a contextual determinant shaping visibility, sampling, and switching costs in agent-mediated markets

### Platform power and agentic gatekeeping

Agentic intermediation may reshape the locus of market power by altering how search and matching are operationalized. In the Anderson–Renault framework, competition depends on the probability that consumers sample competing offers and continue searching. When AI agents operate within vertically integrated platform ecosystems that control ranking logic, data inputs, and transaction routing, the sampling process is no longer decentralized.

If ranking and visibility are mediated through proprietary model architectures, the effective sampling distribution becomes platform-dependent. In economic terms, this can operate as an endogenous modification of search cost and continuation probabilities. Even if technological search costs are low, biased ranking or selective exposure can reduce effective exploration and sustain higher equilibrium prices.

Agentic systems also centralize preference interpretation and transaction routing within the platform’s infrastructure. Because these systems rely on rich behavioral data, purchase histories, browsing patterns, and cross-service signals, they may reinforce incumbents’ informational advantages. In the search-and-differentiation framework, this can increase switching costs and alter entry incentives by changing the expected demand accessible to new entrants.

A further structural dimension of agentic intermediation arises from the accumulation of detailed preference data. As large LLM-based agents repeatedly observe user queries, transactions, and behavioral signals, they construct increasingly refined representations of individual preferences. These representations constitute proprietary informational assets. Switching to alternative agents may entail the loss of accumulated personalization, thereby creating endogenous switching costs and reinforcing platform-level data advantages. In this sense, agentic markets exhibit data-driven increasing returns that may entrench incumbency.

Smaller and newly entering sellers are particularly vulnerable. If ranking mechanisms systematically favor incumbent or affiliated sellers, the sampling probability of competing sellers declines. Since expected demand is proportional to the probability of being sampled, such visibility distortions reduce the expected return to entry and may discourage competitive entry. This reduces the expected payoff from entry and may entrench incumbent sellers even when their products are not superior in price or quality. Thus, gatekeeping modifies not only prices but also the competitive structure of the market.

These dynamics have regulatory implications. The European Union’s Digital Markets Act (DMA) addresses gatekeeper behavior through obligations concerning ranking transparency, non-self-preferencing, and restrictions on cross-service data combination. In agent-mediated environments, such obligations extend beyond interface design to the internal logic of AI-driven recommendation and execution systems. If agentic ranking mechanisms embed monetization incentives or self-preferencing into their sampling logic, the resulting distortions directly affect contestability and entry—central concerns in the economic analysis above.

Figure 4 summarizes how these design levers correspond to the underlying economic mechanisms. Designing marketplaces for AI agents, therefore, requires not only minimizing technical frictions but actively shaping incentives, signal structures, and participation constraints to influence equilibrium outcomes.

## Research opportunities for agentic market theory

Beyond immediate design implications, agentic markets raise several research questions that extend the search-and-differentiation framework developed in this paper.

First, empirical measurement of agentic frictions remains an open challenge. Operationalizing behavioral search costs, signal dilution, and visibility distortions in live marketplaces would allow researchers to estimate how AI-mediated sampling alters equilibrium prices, entry incentives, and welfare outcomes. Developing credible identification strategies for such effects is an important direction for future empirical work.

Second, under what conditions are agentic frictions structural rather than transitional? Improvements in model alignment, robustness, and training objectives (e.g., regret minimization) may reduce certain distortions. However, if frictions arise from probabilistic inference, sampling constraints, or centralized ranking architectures, technological advances may shift rather than eliminate the effective search cost $c_{\text {behavior}}$ or informational noise in match signals.

Third, how do dynamic learning processes among autonomous agents affect equilibrium selection and stability? While our analysis embeds agentic frictions into a static equilibrium model, real-world systems involve repeated interaction, reinforcement learning, and evolving prompt architectures. A central question is whether such dynamics converge toward Nash or coarse correlated equilibria, or instead generate persistent instability and path dependence when behavioral search costs and signal distortions are present.

Finally, the interaction between centralized platform governance and decentralized agent autonomy warrants further study. If platforms control ranking logic, data access, and transaction routing, they effectively shape the sampling distribution and entry payoffs in agent-mediated markets. Understanding how governance rules, transparency requirements, and interoperability constraints influence these primitives is central to assessing long-run market structure.

Together, these questions indicate that agentic markets are not merely an application of existing search theory, but an environment in which core primitives—search cost, informational precision, and entry conditions—become endogenous to algorithmic design and platform architecture.

## Data Availability

No datasets were generated or analyzed during the current study.

## Footnotes

1 See cases by https://www.travelai.com/ or DeltaV by https://fetch.ai/.
